# ERCC1 as a biomarker for bladder cancer patients likely to benefit from adjuvant chemotherapy

**DOI:** 10.1186/1471-2407-12-187

**Published:** 2012-05-22

**Authors:** Jong-Mu Sun, Ji-Youn Sung, Se Hoon Park, Ghee Young Kwon, Byong Chang Jeong, Seong Il Seo, Seong Soo Jeon, Hyun Moo Lee, Jisuk Jo, Han Yong Choi, Ho Yeong Lim

**Affiliations:** 1 Division of Hematology-Oncology, Department of Medicine, Samsung Medical Center, Sungkyunkwan University School of Medicine, Seoul, South Korea; 2 Department of Pathology, Samsung Medical Center, Sungkyunkwan University School of Medicine, Seoul, South Korea; 3 Department of Urology, Samsung Medical Center, Sungkyunkwan University School of Medicine, Seoul, South Korea; 4 Cancer Research Institute, Research Institute for Future Medicine, Samsung Medical Center, Seoul, South Korea

## Abstract

**Background:**

The role of adjuvant chemotherapy and the value of molecular biomarkers in bladder cancer have not been determined. We aimed to assess the predictive and prognostic values of excision repair cross-complementation 1 (ERCC1) in identifying appropriate patients who may potentially benefit from adjuvant chemotherapy for bladder cancer.

**Methods:**

A retrospective analysis was performed on 93 patients with completely resected transitional cell carcinoma of the bladder. ERCC1 expression was assessed by immunohistochemistry. ERCC1 expression was analyzed in 57 patients treated with adjuvant gemcitabine plus cisplatin chemotherapy and 36 who were not treated.

**Results:**

Among 93 patients, ERCC1 expression was positive in 54 (58.1%) and negative in 39 (41.9%). ERCC1 positivity was significantly associated with longer survival (adjusted hazard ratio for death, 0.12, 95% confidence interval [CI] 0.014-0.99; P = 0.049) in the group without adjuvant chemotherapy while ERCC1 positivity was associated with shorter survival among patients who have received adjuvant chemotherapy (adjusted hazard ratio for death, 2.64; 95% CI 1.01-6.85; P = 0.047). Therefore, clinical benefit from adjuvant chemotherapy was associated with ERCC1 negativity as measured by overall survival (test for interaction, P = 0.034) and by disease-free survival (test for interaction, P = 0.20).

**Conclusions:**

Among patients with completely resected transitional cell carcinoma of the bladder, those with ERCC1-negative tumors seemed to benefit more from adjuvant gemcitabine plus cisplatin chemotherapy than those with ERCC1-positive tumors. Future prospective, randomized studies are warranted to confirm our findings.

## Background

Many patients with locally advanced bladder cancer relapse and subsequently die of their disease, even after potentially curative surgery, because of occult micrometastases present at diagnosis. Perioperative chemotherapy has been investigated for patients who undergo cystectomy for locally advanced transitional cell carcinoma of the bladder, and clinical benefit from neoadjuvant cisplatin-based chemotherapy has been demonstrated in several randomized trials [1-3].

Although two meta-analyses showed favorable results for adjuvant chemotherapy for bladder cancer [4,5], no randomized trial has demonstrated the efficacy of adjuvant chemotherapy for overall survival because of small sample size, or early stoppage of patient entry [6-9]. In practice, however, many physicians administer adjuvant chemotherapy despite its weak evidence [10,11]. Further research investigating the effect of adjuvant chemotherapy on bladder cancer survival is of high importance considering its current practice .

Cisplatin is the most important adjuvant chemotherapy agent for bladder cancer and is usually administered with gemcitabine. Its cytotoxicity is attributed to the formation of DNA adducts, which cause inter- and intrastrand cross-linking that inhibits DNA replication. Cisplatin-induced DNA adducts are removed by the nucleotideexcision repair pathway, and the excision repair crosscomplementation 1 (ERCC1) protein is rate-limiting in the nucleotide excision repair pathway. Its increased expression is associated with resistance to cisplatin-based chemotherapy in various tumor types [12-17].

In addition to its predictive role for cisplatin-based chemotherapy, ERCC1 has significant prognostic value because high ERCC1 expression is associated with longer survival in patients who do not receive chemotherapy after complete resection for non-small cell lung cancer [15,18]. ERCC1 has also been supported by studies that demonstrate cancers with extensive genomic alterations have more malignant phenotype and increased growth rates, and ERCC1 may be representative of the intrinsic DNA damage-repair ability of the cell [18,19].

In this study, we sought to determine whether ERCC1 protein expression is an important factor in predicting the clinical outcome of completely resected bladder cancer. To assess the predictive and prognostic value of ERCC1 and to define the subgroup of patients who are most likely to benefit from adjuvant chemotherapy, patients classified by history of adjuvant chemotherapy were analyzed.

## Methods

### Patients and treatment

The initial study population comprised of 137 patients treated with radical cystectomy and bilateral pelvic lymphadenectomy as definitive treatment for clinically localized urothelial cancer of the bladder between January 2004 and December 2010 at Samsung Medical Center (Seoul, Korea). Of these, 93 patients were included in the analysis after excluding 12 patients who had not been completely resected, eight who received neoadjuvant chemotherapy, 17 patients whose tumors are pTa/pT1 with negative node, and seven who did not have available tissue for immunohistochemical analysis for ERCC1. None received pelvic irradiation after complete resection or had documented residual disease before receiving adjuvant chemotherapy. All specimens included in this study were transitional cell carcinoma.

Adjuvant chemotherapy included a maximum of four cycles of gemcitabine (1250 mg/m^2^ on days 1 and 8) plus cisplatin (70 mg/m^2^ on day 1) every 21 days. Adjuvant chemotherapy was given to patients with pathologically advanced bladder cancer (T3/4 or positive node) between three to eight weeks after complete resection. The decision to treat with adjuvant chemotherapy was made after a full discussion with patients, and was based on pathologic tumor stage and patient age or performance status.

Clinicopathologic characteristics including age at operation, sex, histology, tumor stage, and tumor grade were extracted from medical records. The study was approved by the Institutional Review Board of Samsung Medical Center.

### Immunohistochemistry for ERCC1

To accurately assess the median value of ERCC1 in urothelial cancer, we also included additional 149 patients with transitional cell carcinoma originating from completely resected ureter or renal pelvis between January 2004 and December 2010. This resulted in 242 patients for ERCC1 expression analysis.

Representative paraffin blocks, selected by primary evaluation of haematoxylin-eosin stained slides, were chosen for tissue microarray (TMA) preparation. Four tissue cores were collected from each tumor with a sample punch (0.6 mm in diameter) and placed in four new recipient paraffin blocks. Each recipient block contained 100 individual sample tissue cores and three tonsil tissue samples as controls and was prepared with agar in our lab.

Immunohistochemistry was carried out on 4-μm tissue sections using the Bond Polymer Intense Detection System (VisionBioSystems, VIC, Australia) according to the manufacturer's instructions, with minor modifications. In brief, formalin-fixed, paraffin-embedded tissue sections were deparaffinized with Bond Dewax Solution (VisionBioSystems) and an antigen-retrieval procedure performed using Bond ER Solution (VisionBioSystems) for 20 minutes at 100°C. Endogenous peroxidase was quenched by incubation with hydrogen peroxide for 7 minutes. Sections were incubated in a Bond-max automatic slide stainer (VisionBioSystems) for 15 minutes at ambient temperature with primary mouse monoclonal antibody against ERCC1 (1:150; 8 F1, Gene Tex, Irvine, CA, USA) labeled using a biotin-free polymeric horseradish peroxidase (HRP)-linker antibody conjugate system. Bound peroxidase was visualized using a solution of diaminobenzidine as the chromogen, and nuclei were counterstained with Mayer’s hematoxylin. Stromal cells around the tumor portion were used as an internal positive control.

ERCC1 immunohistochemical staining was assessed by two blinded investigators (J Sung and GY Kwon) who reviewed the cases simultaneously at a multihead viewing microscope. Nuclear staining was considered positive. Staining intensity was defined as follows: 0, no staining; 1, weak; 2, moderate; 3, strong. Quantification of positivity (0%-100%) was based on an estimate of the percentage of stained tumor cells in the tissue microarray core. The final histochemical score (H-score) was obtained by multiplying staining intensity by percent positivity, giving H-scores ranging from 0 to 300. An H-score higher than the median was considered positive.

### Statistical analysis

The primary endpoint of overall survival was calculated from the day of operation to the date of death or final follow-up. Disease-free survival was defined from the day of operation to documented diseased recurrence or death from any cause. Patients without recurrent disease at the time of analysis were censored for the final follow-up.

The relationships between ERCC1 expression and clinicopathologic factors were analyzed using a Chi-square test. Survival curves were generated using the Kaplan-Meier method, and the log-rank test was used to compare survival curves according to clinicopathological characteristics including ERCC1 expression. A multivariate regression analysis was carried out using Cox’s regression analysis. A Cox proportional hazards regression model was used to test for interaction between ERCC1 expression and adjuvant chemotherapy. All analyses were performed with the use of SPSS 19.0 (SPSS Inc, Chicago, IL, USA) and SAS 9.1 (SAS Institute Inc, Cary, NC, USA).

## Results

### Patient characteristics

Table 1 lists baseline patient characteristics. The median patient age was 63 years (range, 34–79), and 79 patients (84.9%) had advanced disease (pathologic T3/4 or positive node). Eleven patients had transitional cell carcinoma with squamous differentiation. Of 93 patients who entered the study, 57 (61.3%) were treated with adjuvant chemotherapy and all had advanced disease. Among 36 patients without adjuvant chemotherapy, 22 (61.1%) had advanced disease and did not receive adjuvant chemotherapy because of old age, poor performance, patient refusal, or other causes.

**Table 1:** Characteristics of 93 patients with urothelial bladder carcinoma treated with radical cystecomy and bilateral lymphadenectomy

Characteristic	No. (%)
Total	93 (100.0)
Median age (range)	63 (34 – 79)
Sex	
Men	79 (84.9%)
Women	14 (15.1%)
Pathological T stage	
T1*	4 (4.3%)
T2	15 (16.1%)
T3	52 (55.9%)
T4	22 (23.7%)
Pathological N stage	
Node negative	61 (65.6%)
Node positive	32 (34.4%)
Histologic type	
Squamous differentiation	11 (11.8%)
Other types	82 (88.2%)
Pathological grade†	
G2	10 (10.8%)
G3	83 (89.2%)
ERCC1 expression	
Positive	54 (58.1%)
Negative	39 (41.9%)
Adjuvant chemotherapy (gemcitabine plus cisplatin)	
Yes	57 (61.3%)
4 cycles	24 (25.8%)
3 cycles	28 (30.1%)
2 cycles	4 (4.3%)
1 cycle	1 (1.1%)
No	36 (38.7%)

*all patients have metastatic lymph nodes. † 1973 WHO grading.

### Assessment of ERCC1 expression

**Figure 1:**
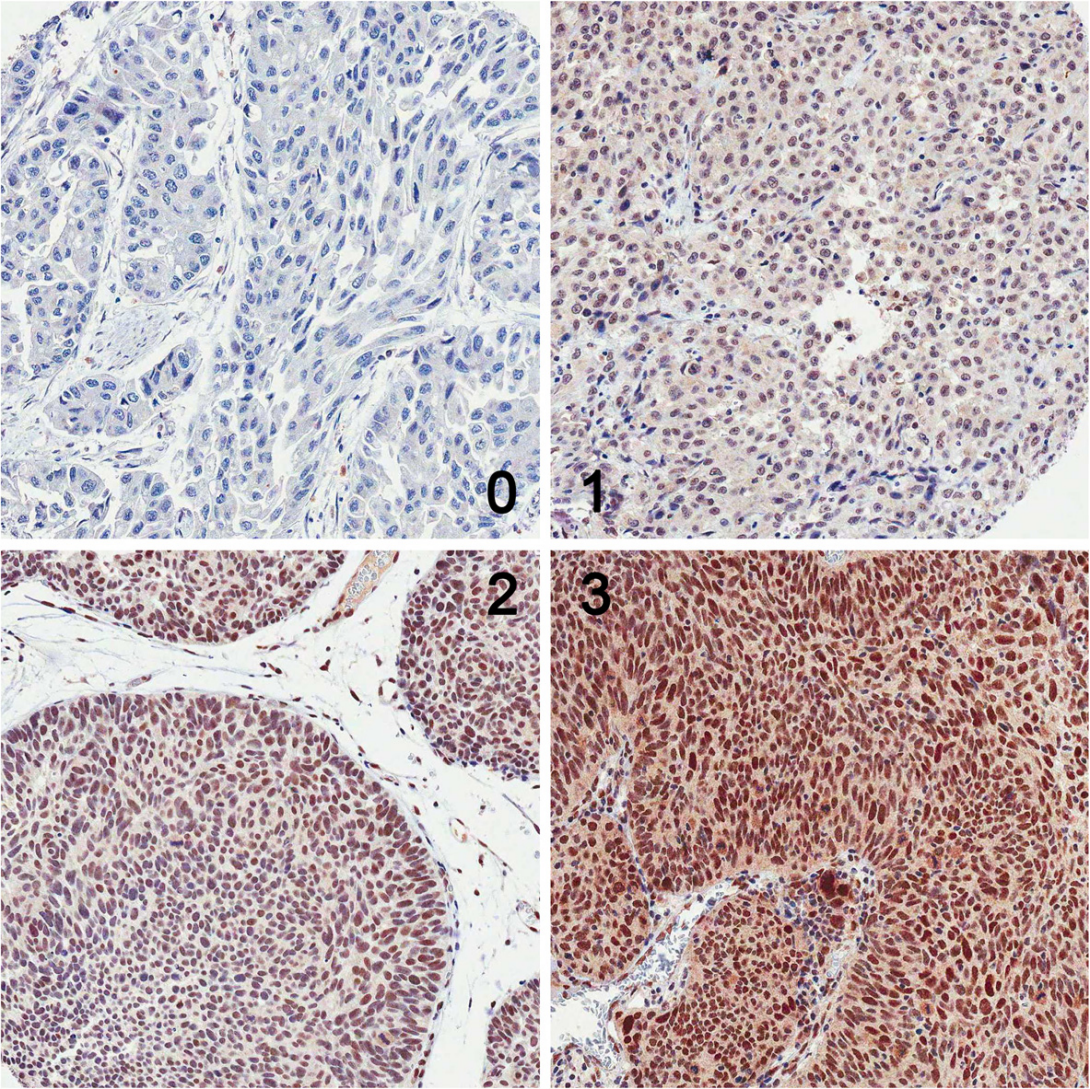
**Representative immunohistochemical staining for ERCC1.** Staining intensity was scored as 0, 1, 2 or 3.

Figure 1 shows that ERCC1 was localized to the nucleus. The median H-score was 50 (range, 0–300). Tumors with an H-score > 50 were deemed ERCC1 positive. Of 93 bladder tumors, 54 (58.1%) were ERCC1 positive and 39 (41.9%) were ERCC1 negative. No significant differences were found in the clinicopathologic parameters between patients with ERCC1-positive and those with ERCC1-negative tumors in groups with or without adjuvant chemotherapy or in the total population (Table 2).

**Table 2:** Associations between ERCC1 expression and clinical characteristics in groups with or without adjuvant chemotherapy

Characteristic	Adjuvant chemotherapy (n = 57)			No adjuvant chemotherapy (n = 36)			Total (n = 93)		
	ERCC1 (+) (n = 34)	ERCC1 (−) (n = 23)	P	ERCC1 (+) (n = 20)	ERCC1 (−) (n = 16)	P	ERCC1 (+) (n = 54)	ERCC1 (−) (n = 39)	P
Sex			0.49			0.19			0.21
Men	29 (85.3%)	18 (78.3%)		19 (95.0%)	13 (81.3%)		48 (88.9%)	31 (79.5%)	
Women	5 (14.7%)	5 (21.7%)		1 (5.0%)	3 (18.8%)		6 (11.1%)	8 (20.5%)	
Age at operation			0.35			0.24			0.92
≥ 65 years	8 (23.5%)	8 (34.8%)		15 (75.0%)	9 (56.3%)		23 (42.6%)	17 (43.6%)	
< 65 years	26 (76.5%)	15 (65.2%)		5 (25.0%)	7 (43.8%)		31 (57.4%)	22 (56.4%)	
T stage			0.68			0.65			0.99
T1-T2	2 (5.9%)	2 (8.7%)		9 (45.0%)	6 (37.5%)		11 (20.4%)	8 (20.5%)	
T3-T4	32 (94.1%)	21 (91.3%)		11 (55.0%)	10 (62.5%)		43 (79.6%)	31 (79.5%)	
N stage			0.48			0.58			0.13
Node negative	16 (47.1%)	13 (56.5%)		16 (80.0%)	16 (100%)		32 (59.3%)	29 (74.4%)	
Node positive	18 (52.9%)	10 (43.5%)		4 (20.0%)	0 (0%)		22 (40.7%)	10 (25.6%)	
Histologic type			0.64			0.87		0.69	
Squamous differentiation									
	6 (17.6%)	3 (13.0%)		1 (5.0%)	1 (6.3%)		7 (13.0%)	4 (10.3%)	
Other types	28 (82.4%)	20 (87.0%)		19 (95.0%)	15 (93.8%)		47 (87.0%)	35 (89.7%)	
Pathologic grade*			0.74			0.69			0.22
Grade 2	2 (5.9%)	5 (21.7%)		2 (10.0%)	1 (6.3%)		6 4 (7.4%)	(15.4%)	
Grade 3	32 (94.1%)	18 (78.3%)		18 (90.0%)	15 (93.8%)		50 (92.6%)	33 (84.6%)	

*1973 WHO grading.

### Overall survival and ERCC1 expression

The 5-year overall survival rate was 56.0% (95% confidence interval [CI], 51.8%–60.3%) for the total study population. According to the Cox model adjusted for the multivariate predictors of survival, ERCC1-positive tumors, compared with ERCC1-negative tumors, had no prognostic value for the entire study population (adjusted hazard ratio [HR] for death, 1.15; 95% CI, 0.55-2.40; P = 0.71).

### Prognostic value of ERCC1 expression according to adjuvant chemotherapy

**Figure 2:**
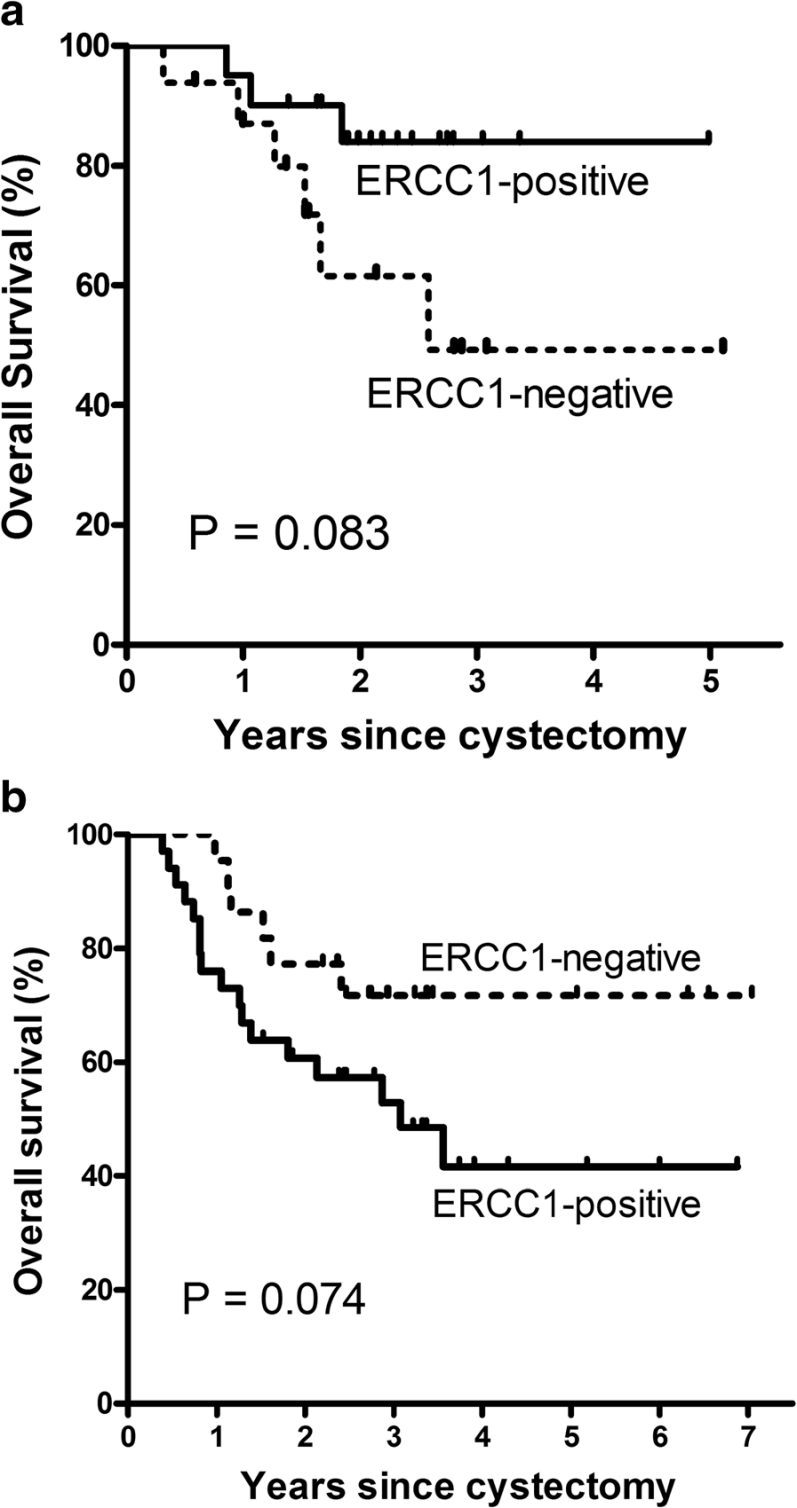
**Overall survival.** Without adjuvant chemotherapy (**a**), with adjuvant chemotherapy (**b**) (interaction P = 0.034).

Among patients without adjuvant chemotherapy, the 5-year overall survival rate was higher in ERCC1-positive tumors than in ERCC1-negative tumors (84.0% vs. 49.2%; P = 0.083) (Table 3, Figure 2a). Other clinical characteristics such as male, positive lymph node, and transitional cell carcinoma with squamous differentiation had poor prognostic values by univariate analysis. Results from multivariate analysis indicate ERCC1 positivity (adjusted HR for death, 0.12; 95% CI 0.014–0.99; P = 0.049), negative lymph node (adjusted HR for death, 0.066; 95% CI 0.005–0.82; P = 0.035), and histologic types other than squamous cell differentiation (adjusted HR for death, 0.033; 95% CI 0.002-0.62; P = 0.022) were significantly associated with longer survival. Among patients who were treated with adjuvant chemotherapy, the 5-year overall survival rate was 41.6% for those with ERCC1-positive tumors and 71.8% for those with ERCC1-negative tumors (P = 0.074) (Table 3, Figure 2b). Multivariate analysis showed that only ERCC1 positivity was significantly associated with shorter survival in the group with adjuvant chemotherapy (adjusted HR for death, 2.64; 95% CI 1.01-6.85; P = 0.047). Overall, the interaction term between ERCC1 expression and adjuvant chemotherapy was significant for overall survival (P = 0.034).

**Table 3:** Overall survival in groups without or with adjuvant chemotherapy

	No adjuvant chemotherapy group			Adjuvant chemotherapy group		
	No. (%)	5-Y OS rate (%)	P	No. (%)	5-Y OS rate (%)	P
All patients	36 (100)	68.3		57 (100)	52.3	
ERCC1 status			0.083			0.074
Positive	20 (55.6)	84.0		34 (59.6)	41.6	
Negative	16 (44.4)	49.2		23 (40.4)	71.8	
Sex			0.080			0.14
Male	32 (88.9)	70.6		47 (82.5)	46.2	
Female	4 (11.1)	50.0		10 (17.5)	77.8	
Age at operation			0.40			0.99
≥ 65 years	24 (66.7)	70.0		16 (28.1)	60.3	
< 65 years	12 (33.3)	61.1		41 (71.9)	51.2	
T stage			0.21			0.57
T1-T2	15 (41.7)	83.6		4 (7.0)	50.0	
T3-T4	21 (58.3)	59.2		53 (93.0)	52.9	
N stage			0.10			0.73
N0	32 (88.9)	70.9		29 (50.9)	47.7	
N1-3	4 (11.1)	50.0		28 (49.1)	59.1	
Histologic type			0.088			0.21
Squamous differentiation	2 (5.6)	50.0		9 (15.8)	77.8	
Other types	34 (94.4)	69.8		48 (84.2)	46.8	
Pathologic Grade*			0.35			0.47
G2	3 (8.3)	100.0		7 (12.3)	71.4	
G3	33 (91.7)	65.7		50 (87.7)	50.2	

*1973 WHO grading.

### Disease-free survival, ERCC1 expression, and adjuvant chemotherapy

**Figure 3:**
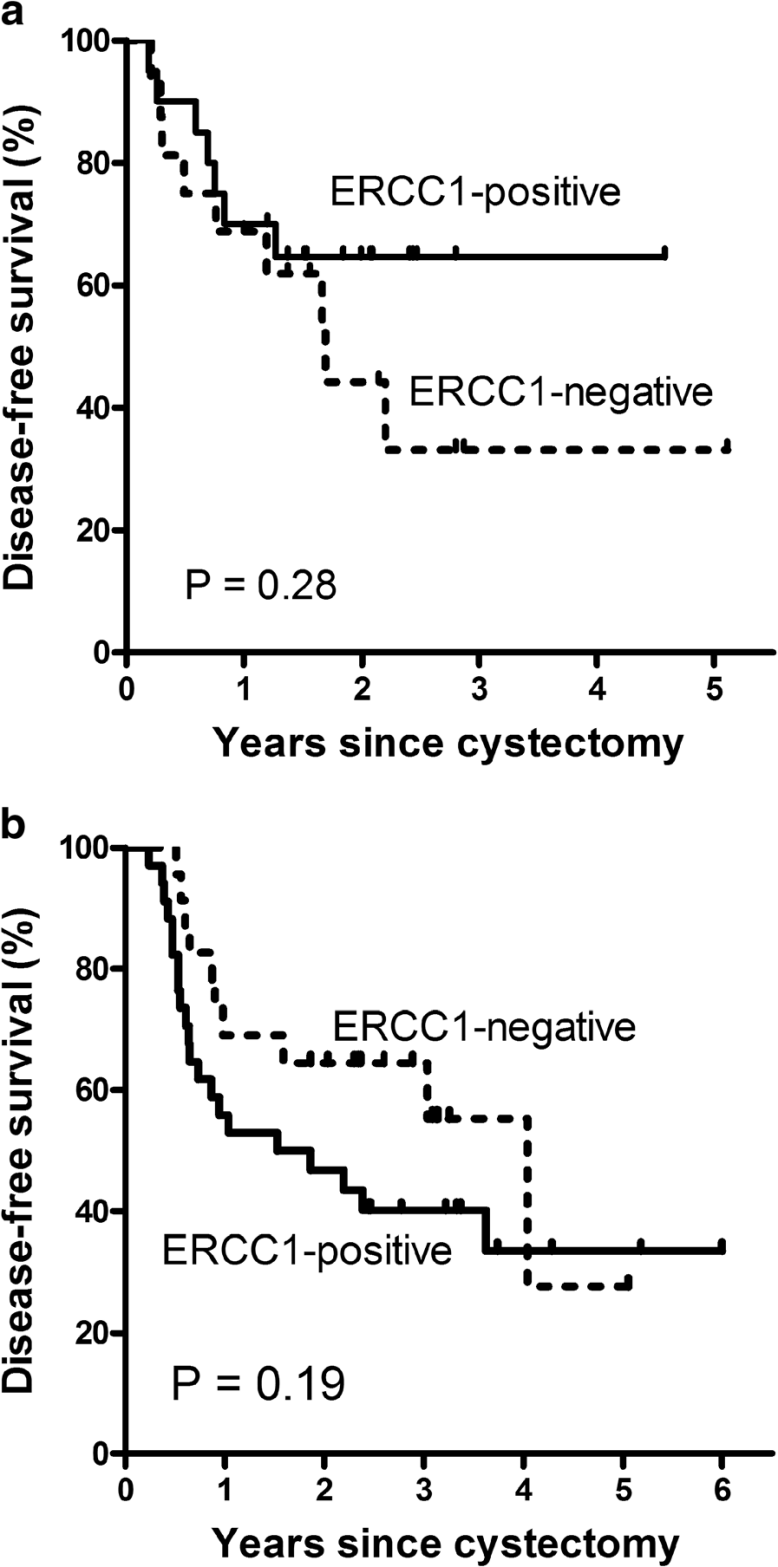
**Disease-free survival.** Without adjuvant chemotherapy (**a**), with adjuvant chemotherapy (**b**) (interaction P = 0.20).

The 2-year disease-free survival rates for ERCC1-positive and ERCC1-negative tumors were 64.6% and 44.2% (P = 0.28) in the group without adjuvant chemotherapy and 46.5% and 64.5% (P = 0.19) in the group with adjuvant chemotherapy, respectively (Figure 3). The interaction term between ERCC1 expression and adjuvant chemotherapy showed borderline significance for disease-free survival (P = 0.20).

## Discussion

In our study, ERCC1 expression provided both prognostic and predictive information in patients with completely resected bladder cancer. Among patients with transitional cell carcinoma of the bladder treated with cystectomy, high tumoral expression of ERCC1 correlated with longer survival in patients without adjuvant chemotherapy and was associated with shorter survival in those with adjuvant chemotherapy. A statistically significant interaction between ERCC1 expression and adjuvant chemotherapy indicated potential benefits of adjuvant chemotherapy in patients with ERCC1-negative tumors.

To date, the role of adjuvant chemotherapy for bladder cancer has been controversial, with no Level 1 evidence supporting adjuvant chemotherapy. In fact, the available data have not demonstrated a clear benefit of adjuvant chemotherapy. Despite mounting evidence favoring neoadjuvant chemotherapy [1-3], physicians are reluctant to adopt its practice as evidenced by only 1.2% of patients with stage III bladder cancer receiving neoadjuvant chemotherapy [10]. Nonetheless, 10.4% of patients from the same cohort received adjuvant chemotherapy, implying a preference for adjuvant chemotherapy over neoadjuvant chemotherapy despite a paucity of evidence [10,11]. Such ubiquitous practice might be attributed to two beliefs: that adjuvant chemotherapy could be given to patients under the most accurate pathologic staging, thereby preventing low-risk patients from unnecessary cytotoxicity; and that up-front surgery increases the chances of curing patients with drug-resistant diseases. In order to strengthen evidence-based practice for adjuvant chemotherapy, further research including rigorous study designs and methodologies are warranted.

A significant strength of this study was the appropriate selection of patients who could potentially benefit from adjuvant chemotherapy based on ERCC1 expression. The target populations in previous adjuvant trials were heterogeneous, ranging from T1-T2 disease to node positive disease, and defined only by pathologic stage. A recent prospective study used p53 expression as a molecular marker for selecting a target population for adjuvant chemotherapy [8]. Patients whose tumors were p53-positive were randomly assigned to adjuvant chemotherapy or to observation, and those with p53-negative tumors were all assigned to observation. Although this trial failed to demonstrate the prognostic and predictive value of p53 due to a high patient refusal rate or lower than expected event rate, the attempt to use a molecular marker in adjuvant chemotherapy is noteworthy. Further molecularly targeted adjuvant chemotherapy should be investigated.

ERCC1 is a component of the nucleotide excision repair pathway, which is essential for the repair of DNA adducts induced by cisplatin-based therapy. Several studies have shown that high ERCC1 expression is a good prognostic factor in patients without cisplatin-based chemotherapy and also a predictor for poor clinical outcome in patients with cisplatin-based chemotherapy for various tumor types [12,14,15,18]. ERCC1 was also previously evaluated in metastatic bladder cancer, and high ERCC1 mRNA or protein expression correlated with poor prognosis in patients treated with cisplatin-based chemotherapy [20-22]. In muscle-invasive bladder cancer, cisplatin-based chemoradiation therapy showed better efficacy for ERCC1-negative tumors than ERCC1-positive tumors [23]. Hoffmann et al. demonstrated that high ERCC1 gene expressions were associated with inferior progression-free survival after cisplatin-based adjuvant chemotherapy for locally advanced bladder cancer [24]. In the present study, the different prognostic values of ERCC1 according to the history of adjuvant gemcitabine plus cisplatin chemotherapy were confirmed in patients with completely resected bladder cancer.

A limitation of our study includes different patient characteristics between two groups with or without adjuvant chemotherapy owing to the retrospective nature of the study. However, such difference and bias which can potentially influence study results were minimized because the value of ERCC1 expression was analyzed independently in each group. In addition, the adjuvant chemotherapy was homogenous with an identical regimen of gemcitabine plus cisplatin.

In summary, we have demonstrated that ERCC1 may potentially be a novel biomarker with clinical predictive and prognostic values in completely resected bladder cancer. Those who have bladder cancer with low ERCC1 expression are more likely to benefit from adjuvant gemcitabine plus cisplatin chemotherapy. Further researches including prospective randomized studies are warranted to confirm our findings.

## Conclusions

ERCC1 expression has a different prognostic nature according to the history of adjuvant chemotherapy: ERCC1-positivity is associated with poorer prognosis in the group with adjuvant chemotherapy while it has better prognosis in those without adjuvant chemotherapy. In bladder cancer, the relevance of ERCC1 expression as a biomarker selecting patients for adjuvant chemotherapy should be confirmed in further prospective studies.

## Misc

Jong-Mu Sun and Ji-Youn Sung contributed equally to this work.

## Competing interests

All authors declare that they have no actual or potential competing interests including any financial, personal or other relationships with other people or companies/organizations that could inappropriately influence this article.

## Authors’ contributions

JS contributed to the design, acquisition of data, analysis of data, and drafting the manuscript. JS and GYK contributed to the conception, pathologic review, and drafted the manuscript. HYL contributed to the conception, design, and provided final approval of the version to be published. JJ contributed to the analysis of data. SHP, BCJ, SIS, SSJ, HML, and HYC contributed to the acquisition of data. All authors read and approved the final manuscript.

## Pre-publication history

The pre-publication history for this paper can be accessed here:

http://www.biomedcentral.com/1471-2407/12/187/prepub
